# 4-(1-Cyclo­propyl-6-fluoro-4-oxo-1,4-dihydro­quinolin-7-yl)piperazin-1-ium 2,4,5-tricarb­oxy­benzene-1-carboxyl­ate monohydrate

**DOI:** 10.1107/S1600536812008392

**Published:** 2012-02-29

**Authors:** Shi-Wei Yan, Yan-Chen Liang, Qin Liao, Guang-Hua Xin, Zhong-Li Ye

**Affiliations:** a College of Chemistry and Chemical Engineering, Southwest University, Chongqing 400715, People’s Republic of China

## Abstract

In the crystal of title compound, C_16_H_19_FN_3_O^+^·C_10_H_5_O_8_^−^·H_2_O, the water mol­ecule and the ions are connected by inter­molecular N—H⋯O and O—H⋯O hydrogen bonds and π–π stacking [centroid–centroid separation = 3.602 (1) Å] between the benzene ring and the pyridine ring, generating a three-dimensional supra­molecular structure.

## Related literature

For general background on the use of quinolones in the treatment of infections, see: Barbas *et al.* (2006 ▶); Basavoju *et al.* (2006 ▶); Xiao *et al.* (2005 ▶).
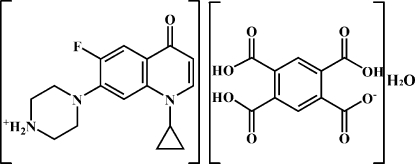


## Experimental

### 

#### Crystal data

C_16_H_19_FN_3_O^+^·C_10_H_5_O_8_^−^·H_2_O
*M* *_r_* = 559.50
Triclinic, 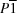

*a* = 9.5537 (19) Å
*b* = 11.300 (2) Å
*c* = 11.686 (2) Å
α = 77.03 (3)°
β = 87.01 (3)°
γ = 88.65 (3)°
*V* = 1227.7 (4) Å^3^
*Z* = 2
Mo *K*α radiation
μ = 0.12 mm^−1^
*T* = 293 K
0.40 × 0.35 × 0.32 mm

#### Data collection

Bruker APEX CCD diffractometer
Absorption correction: multi-scan (*SADABS*; Sheldrick, 1996 ▶) *T*_min_ = 0.953, *T*_max_ = 0.962
12113 measured reflections
5561 independent reflections
3880 reflections with *I* > 2σ(*I*)
*R*_int_ = 0.024

#### Refinement

*R*[*F* ^2^ > 2σ(*F* ^2^)] = 0.050
*wR*(*F* ^2^) = 0.159
*S* = 0.99
5561 reflections
373 parameters
1 restraint
H atoms treated by a mixture of independent and constrained refinement
Δρ_max_ = 0.31 e Å^−3^
Δρ_min_ = −0.31 e Å^−3^

### 

Data collection: *SMART* (Bruker, 2001 ▶); cell refinement: *SAINT* (Bruker, 2001 ▶); data reduction: *SAINT*; program(s) used to solve structure: *SHELXS97* (Sheldrick, 2008 ▶); program(s) used to refine structure: *SHELXL97* (Sheldrick, 2008 ▶); molecular graphics: *SHELXTL-Plus* (Sheldrick, 2008 ▶); software used to prepare material for publication: *SHELXL97*.

## Supplementary Material

Crystal structure: contains datablock(s) I, global. DOI: 10.1107/S1600536812008392/ff2056sup1.cif

Structure factors: contains datablock(s) I. DOI: 10.1107/S1600536812008392/ff2056Isup2.hkl

Supplementary material file. DOI: 10.1107/S1600536812008392/ff2056Isup3.cml

Additional supplementary materials:  crystallographic information; 3D view; checkCIF report

## Figures and Tables

**Table 1:** Hydrogen-bond geometry (Å, °)

*D*—H⋯*A*	*D*—H	H⋯*A*	*D*⋯*A*	*D*—H⋯*A*
O1—H1*C*⋯O3^i^	0.94	1.58	2.5170 (18)	177
O1—H1*C*⋯O4^i^	0.94	2.52	3.078 (2)	118
N1—H1*B*⋯O3^i^	0.93 (3)	2.54 (3)	3.006 (3)	111 (2)
N1—H1*B*⋯O1*W*^ii^	0.93 (3)	2.04 (3)	2.929 (3)	158 (2)
O8—H8*A*⋯O9^iii^	1.00	1.52	2.519 (2)	178.2

Symmetry codes: (i) 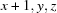; (ii) 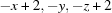; (iii) 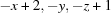.

**Fig. 1.**
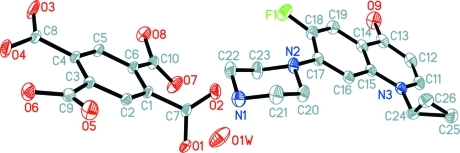
The structure of 1. Displacement ellipsoids are drawn at the 30% probability level.

## supplementary crystallographic information

### Comment

Ciprofloxacin (cipH,1-cyclopropyl-6-fluoro-1,4-dihydro-4-oxo-7-(1-piperazinyl)-3-quinoline carboxylic acid) is member of a class of quinolones used to treat infections (Xiao *et al.* 2005; Barbas *et al.* 2006; Basavoju *et al.* 2006). In this paper, we synthesized the complex of L from cipH ligand, and then obtained the compound 1 by reaction with 1,2,4,5-H_4_betc in hydrothermal conditions. Here we report the title compound 1.

As shown in Fig. 1, compound 1 contains one [HL]^+^ cation, one [1,2,4-H_3_betc]^-^ anion and one H_2_O molecule in the asymmetric unit. Then the molecules and the ions are further linked by intermolecular N—H···O and O—H···O hydrogen-bonding interactions (Table 1) and π–π stacking (separation 3.602 (1) Å) between the benzene rings and the pyridine rings to form a three-dimensional supramolecular structure.

### Experimental

A mixture of Mn(CH_3_COO)_2_.4H_2_O(0.123 g, 0.5 mmol), cipH (0.192 g, 0.5 mmol), 1,2,4,5-H_4_betc (0.127, 0.5 mmol) and distilled water (7 ml) was stirred for 20 min in air. The mixture was then transferred to a 17 ml Teflon-lined hydrothermal bomb. The bomb was kept at 423 K for 129 h under autogenous pressure. Upon cooling, colorless block of 1 was obtained from the reaction mixture.

### Refinement

The H atoms bonded to C atoms were positioned geometrically and refined using a riding model approximation [aromatic C—H = 0.93 Å, aliphatic C—H = 0.97 Å], with *U*_iso_(H) = 1.2 *U*_eq_(C). The H on N atoms were located in a difference Fourier map, and refined with *U*_iso_(H) = 1.3 and 1.4*U*_eq_(N). The H atoms bonded to O atoms were located in a difference Fourier maps and with *U*_iso_(H) = 1.2 and 1.5*U*_eq_(O) for carboxyl groups of [HL]^+^ cation and [1,2,4-H_3_betc]^-^ anion and with *U*_iso_(H) = 1.2*U*_eq_(OW) for H_2_O molecule, respectively.

### Crystal data

**Table:**

C_16_H_19_FN_3_O^+^·C_10_H_5_O_8_^−^·H_2_O	*Z* = 2
*M**_r_* = 559.50	*F*(000) = 584
Triclinic, *P*1	*D*_x_ = 1.514 Mg m^−^^3^
*a* = 9.5537 (19) Å	Mo *K*α radiation, λ = 0.71073 Å
*b* = 11.300 (2) Å	Cell parameters from 12113 reflections
*c* = 11.686 (2) Å	θ = 3.0–27.5°
α = 77.03 (3)°	µ = 0.12 mm^−^^1^
β = 87.01 (3)°	*T* = 293 K
γ = 88.65 (3)°	Block, colourless
*V* = 1227.7 (4) Å^3^	0.40 × 0.35 × 0.32 mm

### Data collection

**Table:**

Bruker APEX CCD diffractometer	5561 independent reflections
Radiation source: fine-focus sealed tube	3880 reflections with *I* > 2σ(*I*)
Graphite monochromator	*R*_int_ = 0.024
φ and ω scans	θ_max_ = 27.5°, θ_min_ = 3.0°
Absorption correction: multi-scan (*SADABS*; Sheldrick, 1996)	*h* = −12→11
*T*_min_ = 0.953, *T*_max_ = 0.962	*k* = −13→14
12113 measured reflections	*l* = −15→15

### Refinement

**Table:**

Refinement on *F*^2^	Primary atom site location: structure-invariant direct methods
Least-squares matrix: full	Secondary atom site location: difference Fourier map
*R*[*F*^2^ > 2σ(*F*^2^)] = 0.050	Hydrogen site location: inferred from neighbouring sites
*wR*(*F*^2^) = 0.159	H atoms treated by a mixture of independent and constrained refinement
*S* = 0.99	*w* = 1/[σ^2^(*F*_o_^2^) + (0.110*P*)^2^] where *P* = (*F*_o_^2^ + 2*F*_c_^2^)/3
5561 reflections	(Δ/σ)_max_ < 0.001
373 parameters	Δρ_max_ = 0.31 e Å^−^^3^
1 restraint	Δρ_min_ = −0.31 e Å^−^^3^

### Special details

**Table:**

Geometry. All e.s.d.'s (except the e.s.d. in the dihedral angle between two l.s. planes) are estimated using the full covariance matrix. The cell e.s.d.'s are taken into account individually in the estimation of e.s.d.'s in distances, angles and torsion angles; correlations between e.s.d.'s in cell parameters are only used when they are defined by crystal symmetry. An approximate (isotropic) treatment of cell e.s.d.'s is used for estimating e.s.d.'s involving l.s. planes.
Refinement. Refinement of *F*^2^ against ALL reflections. The weighted *R*-factor *wR* and goodness of fit *S* are based on *F*^2^, conventional *R*-factors *R* are based on *F*, with *F* set to zero for negative *F*^2^. The threshold expression of *F*^2^ > σ(*F*^2^) is used only for calculating *R*-factors(gt) *etc*. and is not relevant to the choice of reflections for refinement. *R*-factors based on *F*^2^ are statistically about twice as large as those based on *F*, and *R*-factors based on ALL data will be even larger.

### Fractional atomic coordinates and isotropic or equivalent isotropic displacement parameters (Å^2^)

**Table:**

	*x*	*y*	*z*	*U*_iso_*/*U*_eq_	
C1	0.60114 (14)	0.33816 (15)	1.07824 (14)	0.0295 (3)	
N1	1.01985 (19)	0.2831 (2)	0.83946 (17)	0.0547 (5)	
H1A	0.957 (3)	0.321 (3)	0.878 (3)	0.079 (8)*	
H1B	1.069 (3)	0.228 (3)	0.895 (3)	0.075 (8)*	
O1	0.82943 (12)	0.29979 (18)	1.14009 (13)	0.0685 (5)	
H1C	0.9252	0.3009	1.1161	0.082*	
O1W	0.87507 (18)	−0.06407 (16)	1.0022 (2)	0.0926 (8)	
H1WA	0.8177	−0.0216	0.9557	0.111*	
H1WB	0.7853	−0.0240	1.0206	0.111*	
F1	1.02031 (11)	0.14064 (13)	0.45876 (13)	0.0624 (4)	
C2	0.54681 (15)	0.41608 (15)	1.14539 (14)	0.0299 (3)	
H2A	0.6083	0.4647	1.1729	0.036*	
N2	1.14351 (15)	0.24609 (15)	0.62194 (15)	0.0415 (4)	
O2	0.80487 (12)	0.37949 (13)	0.95197 (12)	0.0477 (3)	
C3	0.40408 (15)	0.42509 (14)	1.17374 (14)	0.0287 (3)	
N3	1.59322 (15)	0.17920 (14)	0.42816 (13)	0.0380 (3)	
O3	0.08715 (12)	0.3036 (2)	1.08262 (15)	0.0738 (6)	
C4	0.31204 (14)	0.35278 (15)	1.12896 (14)	0.0292 (3)	
O4	0.09600 (12)	0.37950 (14)	1.23777 (13)	0.0506 (4)	
C5	0.36736 (15)	0.27751 (15)	1.05850 (15)	0.0309 (3)	
H5A	0.3061	0.2322	1.0267	0.037*	
O5	0.45510 (15)	0.59231 (14)	1.25440 (14)	0.0538 (4)	
C6	0.51010 (14)	0.26699 (14)	1.03351 (14)	0.0289 (3)	
O6	0.25111 (14)	0.50869 (15)	1.31073 (13)	0.0511 (4)	
H6A	0.190 (3)	0.457 (3)	1.282 (3)	0.080 (9)*	
C7	0.75666 (15)	0.33869 (15)	1.05065 (15)	0.0322 (4)	
O7	0.68669 (13)	0.13815 (14)	0.97773 (15)	0.0549 (4)	
C8	0.15442 (16)	0.34709 (17)	1.15168 (16)	0.0379 (4)	
O8	0.47677 (13)	0.13899 (13)	0.90410 (13)	0.0462 (3)	
H8A	0.5250	0.0896	0.8522	0.055*	
C9	0.36945 (17)	0.51692 (16)	1.24860 (15)	0.0360 (4)	
O9	1.40033 (17)	−0.01196 (15)	0.22317 (16)	0.0628 (4)	
C10	0.56713 (16)	0.17555 (16)	0.96764 (16)	0.0349 (4)	
C11	1.66801 (19)	0.12425 (18)	0.35279 (18)	0.0437 (4)	
H11A	1.7651	0.1308	0.3486	0.052*	
C12	1.6091 (2)	0.06014 (18)	0.28300 (19)	0.0472 (5)	
H12A	1.6662	0.0252	0.2324	0.057*	
C13	1.4631 (2)	0.04559 (17)	0.28592 (18)	0.0438 (4)	
C14	1.38275 (19)	0.10197 (15)	0.36840 (16)	0.0376 (4)	
C15	1.44897 (17)	0.16657 (15)	0.43999 (15)	0.0347 (4)	
C16	1.36961 (17)	0.21379 (16)	0.52447 (15)	0.0357 (4)	
H16A	1.4151	0.2539	0.5729	0.043*	
C17	1.22544 (18)	0.20214 (16)	0.53736 (16)	0.0366 (4)	
C18	1.16172 (19)	0.14494 (17)	0.45746 (18)	0.0421 (4)	
C19	1.2362 (2)	0.09473 (17)	0.37802 (18)	0.0430 (4)	
H19A	1.1897	0.0553	0.3296	0.052*	
C20	1.21999 (19)	0.3099 (2)	0.6953 (2)	0.0491 (5)	
H20A	1.2804	0.3702	0.6455	0.059*	
H20B	1.2785	0.2524	0.7469	0.059*	
C21	1.1188 (2)	0.3715 (2)	0.7680 (2)	0.0586 (6)	
H21A	1.1708	0.4092	0.8192	0.070*	
H21B	1.0670	0.4348	0.7164	0.070*	
C22	0.9432 (2)	0.2206 (2)	0.7649 (2)	0.0552 (5)	
H22A	0.8870	0.2789	0.7119	0.066*	
H22B	0.8808	0.1613	0.8136	0.066*	
C23	1.0456 (2)	0.1578 (2)	0.6948 (2)	0.0546 (5)	
H23A	1.0979	0.0963	0.7479	0.065*	
H23B	0.9948	0.1179	0.6447	0.065*	
C24	1.66395 (19)	0.25073 (19)	0.49675 (17)	0.0438 (4)	
H24A	1.6722	0.2135	0.5804	0.053*	
C25	1.7802 (2)	0.3311 (2)	0.4393 (2)	0.0533 (5)	
H25A	1.8061	0.3323	0.3577	0.064*	
H25B	1.8575	0.3412	0.4868	0.064*	
C26	1.6408 (2)	0.3841 (2)	0.46637 (19)	0.0499 (5)	
H26A	1.6338	0.4263	0.5302	0.060*	
H26B	1.5824	0.4174	0.4011	0.060*	

### Atomic displacement parameters (Å^2^)

**Table:**

	*U*^11^	*U*^22^	*U*^33^	*U*^12^	*U*^13^	*U*^23^
C1	0.0195 (7)	0.0390 (9)	0.0314 (8)	0.0013 (6)	0.0018 (6)	−0.0117 (7)
N1	0.0458 (9)	0.0744 (13)	0.0467 (10)	0.0155 (9)	0.0116 (8)	−0.0240 (9)
O1	0.0156 (6)	0.1473 (17)	0.0407 (8)	0.0011 (7)	−0.0006 (5)	−0.0176 (9)
O1W	0.0558 (10)	0.0569 (10)	0.167 (2)	0.0104 (8)	−0.0303 (12)	−0.0242 (12)
F1	0.0350 (6)	0.0850 (10)	0.0770 (9)	0.0021 (5)	0.0012 (6)	−0.0402 (8)
C2	0.0227 (7)	0.0405 (9)	0.0295 (8)	−0.0016 (6)	−0.0016 (6)	−0.0144 (7)
N2	0.0365 (7)	0.0475 (9)	0.0444 (9)	−0.0012 (6)	0.0147 (6)	−0.0220 (7)
O2	0.0322 (6)	0.0623 (9)	0.0429 (8)	0.0087 (5)	0.0136 (5)	−0.0043 (6)
C3	0.0246 (7)	0.0355 (8)	0.0275 (8)	0.0041 (6)	−0.0008 (6)	−0.0108 (6)
N3	0.0354 (7)	0.0431 (8)	0.0340 (8)	0.0071 (6)	0.0057 (6)	−0.0081 (6)
O3	0.0160 (6)	0.1546 (18)	0.0704 (11)	−0.0076 (7)	0.0026 (6)	−0.0669 (12)
C4	0.0176 (6)	0.0395 (9)	0.0316 (8)	0.0033 (5)	−0.0006 (6)	−0.0105 (6)
O4	0.0260 (6)	0.0772 (10)	0.0547 (9)	−0.0001 (6)	0.0115 (6)	−0.0310 (7)
C5	0.0216 (7)	0.0376 (9)	0.0372 (9)	−0.0014 (6)	−0.0012 (6)	−0.0159 (7)
O5	0.0540 (8)	0.0583 (9)	0.0593 (9)	−0.0052 (6)	0.0054 (7)	−0.0361 (7)
C6	0.0212 (7)	0.0349 (8)	0.0334 (8)	0.0022 (5)	−0.0002 (6)	−0.0144 (7)
O6	0.0396 (7)	0.0739 (10)	0.0494 (9)	0.0046 (6)	0.0076 (6)	−0.0366 (7)
C7	0.0210 (7)	0.0424 (9)	0.0364 (9)	−0.0012 (6)	0.0038 (6)	−0.0167 (7)
O7	0.0311 (6)	0.0642 (9)	0.0816 (11)	0.0138 (6)	−0.0027 (7)	−0.0437 (8)
C8	0.0195 (7)	0.0567 (11)	0.0392 (9)	0.0029 (6)	0.0013 (7)	−0.0151 (8)
O8	0.0384 (7)	0.0542 (8)	0.0573 (9)	0.0032 (5)	−0.0023 (6)	−0.0364 (7)
C9	0.0340 (8)	0.0448 (10)	0.0327 (9)	0.0076 (7)	−0.0015 (7)	−0.0171 (7)
O9	0.0694 (10)	0.0643 (10)	0.0674 (11)	−0.0015 (7)	0.0143 (8)	−0.0453 (8)
C10	0.0273 (8)	0.0390 (9)	0.0419 (10)	0.0000 (6)	0.0039 (7)	−0.0175 (7)
C11	0.0379 (9)	0.0470 (11)	0.0423 (10)	0.0107 (7)	0.0119 (8)	−0.0061 (8)
C12	0.0565 (11)	0.0414 (10)	0.0427 (10)	0.0112 (8)	0.0163 (9)	−0.0131 (8)
C13	0.0570 (11)	0.0350 (9)	0.0401 (10)	0.0060 (8)	0.0099 (8)	−0.0135 (8)
C14	0.0454 (9)	0.0326 (9)	0.0358 (9)	0.0054 (7)	0.0068 (7)	−0.0123 (7)
C15	0.0381 (8)	0.0332 (9)	0.0308 (8)	0.0058 (6)	0.0064 (7)	−0.0054 (7)
C16	0.0368 (9)	0.0403 (9)	0.0310 (9)	0.0037 (7)	0.0058 (7)	−0.0123 (7)
C17	0.0393 (9)	0.0344 (9)	0.0367 (9)	0.0040 (6)	0.0086 (7)	−0.0120 (7)
C18	0.0361 (9)	0.0446 (10)	0.0479 (11)	0.0033 (7)	0.0034 (8)	−0.0168 (8)
C19	0.0466 (10)	0.0422 (10)	0.0449 (11)	0.0000 (7)	0.0021 (8)	−0.0208 (8)
C20	0.0393 (9)	0.0599 (12)	0.0554 (12)	−0.0029 (8)	0.0130 (8)	−0.0313 (10)
C21	0.0584 (12)	0.0630 (14)	0.0634 (14)	0.0004 (10)	0.0168 (10)	−0.0375 (11)
C22	0.0406 (10)	0.0732 (15)	0.0520 (12)	0.0010 (9)	0.0169 (9)	−0.0193 (11)
C23	0.0468 (10)	0.0613 (13)	0.0588 (13)	−0.0067 (9)	0.0204 (9)	−0.0248 (11)
C24	0.0357 (9)	0.0642 (12)	0.0312 (9)	0.0042 (8)	−0.0013 (7)	−0.0106 (8)
C25	0.0429 (10)	0.0704 (14)	0.0481 (12)	−0.0046 (9)	0.0033 (9)	−0.0170 (10)
C26	0.0488 (10)	0.0632 (13)	0.0442 (11)	0.0030 (9)	−0.0028 (9)	−0.0261 (10)

### Geometric parameters (Å, º)

**Table:**

C1—C2	1.381 (2)	O8—C10	1.299 (2)
C1—C6	1.395 (2)	O8—H8A	1.0011
C1—C7	1.504 (2)	O9—C13	1.264 (2)
N1—C22	1.471 (3)	C11—C12	1.356 (3)
N1—C21	1.476 (3)	C11—H11A	0.9300
N1—H1A	0.89 (3)	C12—C13	1.406 (3)
N1—H1B	0.93 (3)	C12—H12A	0.9300
O1—C7	1.274 (2)	C13—C14	1.449 (3)
O1—H1C	0.9423	C14—C19	1.402 (3)
O1W—H1WA	0.8534	C14—C15	1.409 (3)
O1W—H1WB	0.9965	C15—C16	1.405 (2)
F1—C18	1.352 (2)	C16—C17	1.384 (2)
C2—C3	1.393 (2)	C16—H16A	0.9300
C2—H2A	0.9300	C17—C18	1.417 (3)
N2—C17	1.399 (2)	C18—C19	1.356 (3)
N2—C20	1.468 (3)	C19—H19A	0.9300
N2—C23	1.475 (3)	C20—C21	1.513 (3)
O2—C7	1.213 (2)	C20—H20A	0.9700
C3—C4	1.410 (2)	C20—H20B	0.9700
C3—C9	1.521 (2)	C21—H21A	0.9700
N3—C11	1.353 (2)	C21—H21B	0.9700
N3—C15	1.385 (2)	C22—C23	1.508 (3)
N3—C24	1.458 (2)	C22—H22A	0.9700
O3—C8	1.246 (2)	C22—H22B	0.9700
C4—C5	1.389 (2)	C23—H23A	0.9700
C4—C8	1.516 (2)	C23—H23B	0.9700
O4—C8	1.247 (2)	C24—C26	1.483 (3)
C5—C6	1.388 (2)	C24—C25	1.485 (3)
C5—H5A	0.9300	C24—H24A	0.9800
O5—C9	1.210 (2)	C25—C26	1.495 (3)
C6—C10	1.498 (2)	C25—H25A	0.9700
O6—C9	1.305 (2)	C25—H25B	0.9700
O6—H6A	0.96 (3)	C26—H26A	0.9700
O7—C10	1.211 (2)	C26—H26B	0.9700
			
C2—C1—C6	119.26 (13)	N3—C15—C16	120.88 (16)
C2—C1—C7	118.20 (14)	N3—C15—C14	119.00 (16)
C6—C1—C7	122.45 (14)	C16—C15—C14	120.09 (16)
C22—N1—C21	110.98 (18)	C17—C16—C15	121.64 (17)
C22—N1—H1A	108.0 (18)	C17—C16—H16A	119.2
C21—N1—H1A	110.1 (19)	C15—C16—H16A	119.2
C22—N1—H1B	111.1 (18)	C16—C17—N2	123.13 (17)
C21—N1—H1B	109.4 (18)	C16—C17—C18	116.46 (16)
H1A—N1—H1B	107 (3)	N2—C17—C18	120.39 (16)
C7—O1—H1C	109.4	F1—C18—C19	117.83 (17)
H1WA—O1W—H1WB	50.5	F1—C18—C17	119.17 (16)
C1—C2—C3	122.95 (14)	C19—C18—C17	122.99 (17)
C1—C2—H2A	118.5	C18—C19—C14	120.40 (18)
C3—C2—H2A	118.5	C18—C19—H19A	119.8
C17—N2—C20	115.57 (14)	C14—C19—H19A	119.8
C17—N2—C23	115.15 (15)	N2—C20—C21	110.56 (16)
C20—N2—C23	110.83 (17)	N2—C20—H20A	109.5
C2—C3—C4	117.79 (14)	C21—C20—H20A	109.5
C2—C3—C9	113.40 (14)	N2—C20—H20B	109.5
C4—C3—C9	128.79 (13)	C21—C20—H20B	109.5
C11—N3—C15	119.49 (16)	H20A—C20—H20B	108.1
C11—N3—C24	120.26 (15)	N1—C21—C20	110.75 (19)
C15—N3—C24	120.26 (15)	N1—C21—H21A	109.5
C5—C4—C3	118.78 (13)	C20—C21—H21A	109.5
C5—C4—C8	115.23 (14)	N1—C21—H21B	109.5
C3—C4—C8	125.98 (15)	C20—C21—H21B	109.5
C6—C5—C4	122.83 (14)	H21A—C21—H21B	108.1
C6—C5—H5A	118.6	N1—C22—C23	109.77 (17)
C4—C5—H5A	118.6	N1—C22—H22A	109.7
C5—C6—C1	118.33 (14)	C23—C22—H22A	109.7
C5—C6—C10	121.34 (13)	N1—C22—H22B	109.7
C1—C6—C10	120.18 (13)	C23—C22—H22B	109.7
C9—O6—H6A	108.6 (19)	H22A—C22—H22B	108.2
O2—C7—O1	124.70 (14)	N2—C23—C22	110.56 (18)
O2—C7—C1	121.15 (15)	N2—C23—H23A	109.5
O1—C7—C1	114.03 (15)	C22—C23—H23A	109.5
O3—C8—O4	121.84 (15)	N2—C23—H23B	109.5
O3—C8—C4	116.41 (16)	C22—C23—H23B	109.5
O4—C8—C4	121.68 (15)	H23A—C23—H23B	108.1
C10—O8—H8A	110.2	N3—C24—C26	117.23 (15)
O5—C9—O6	121.09 (17)	N3—C24—C25	118.92 (17)
O5—C9—C3	119.46 (16)	C26—C24—C25	60.50 (15)
O6—C9—C3	119.33 (16)	N3—C24—H24A	116.2
O7—C10—O8	124.51 (17)	C26—C24—H24A	116.2
O7—C10—C6	121.15 (15)	C25—C24—H24A	116.2
O8—C10—C6	114.30 (13)	C24—C25—C26	59.67 (14)
N3—C11—C12	123.56 (17)	C24—C25—H25A	117.8
N3—C11—H11A	118.2	C26—C25—H25A	117.8
C12—C11—H11A	118.2	C24—C25—H25B	117.8
C11—C12—C13	121.11 (18)	C26—C25—H25B	117.8
C11—C12—H12A	119.4	H25A—C25—H25B	114.9
C13—C12—H12A	119.4	C24—C26—C25	59.83 (14)
O9—C13—C12	124.86 (18)	C24—C26—H26A	117.8
O9—C13—C14	119.59 (18)	C25—C26—H26A	117.8
C12—C13—C14	115.55 (18)	C24—C26—H26B	117.8
C19—C14—C15	118.14 (16)	C25—C26—H26B	117.8
C19—C14—C13	120.63 (17)	H26A—C26—H26B	114.9
C15—C14—C13	121.23 (17)		
			
C6—C1—C2—C3	1.8 (3)	C12—C13—C14—C15	0.0 (3)
C7—C1—C2—C3	178.42 (14)	C11—N3—C15—C16	174.60 (16)
C1—C2—C3—C4	−1.5 (2)	C24—N3—C15—C16	−5.1 (2)
C1—C2—C3—C9	179.95 (15)	C11—N3—C15—C14	−3.4 (2)
C2—C3—C4—C5	−0.6 (2)	C24—N3—C15—C14	176.89 (15)
C9—C3—C4—C5	177.76 (16)	C19—C14—C15—N3	−177.24 (16)
C2—C3—C4—C8	178.52 (15)	C13—C14—C15—N3	2.0 (2)
C9—C3—C4—C8	−3.2 (3)	C19—C14—C15—C16	4.8 (3)
C3—C4—C5—C6	2.4 (3)	C13—C14—C15—C16	−175.96 (16)
C8—C4—C5—C6	−176.81 (15)	N3—C15—C16—C17	179.91 (16)
C4—C5—C6—C1	−2.1 (3)	C14—C15—C16—C17	−2.1 (3)
C4—C5—C6—C10	173.58 (16)	C15—C16—C17—N2	178.80 (16)
C2—C1—C6—C5	0.0 (2)	C15—C16—C17—C18	−2.7 (3)
C7—C1—C6—C5	−176.49 (15)	C20—N2—C17—C16	1.9 (3)
C2—C1—C6—C10	−175.70 (15)	C23—N2—C17—C16	−129.4 (2)
C7—C1—C6—C10	7.8 (3)	C20—N2—C17—C18	−176.54 (18)
C2—C1—C7—O2	−109.5 (2)	C23—N2—C17—C18	52.2 (2)
C6—C1—C7—O2	67.1 (2)	C16—C17—C18—F1	−174.11 (17)
C2—C1—C7—O1	66.7 (2)	N2—C17—C18—F1	4.4 (3)
C6—C1—C7—O1	−116.7 (2)	C16—C17—C18—C19	5.2 (3)
C5—C4—C8—O3	−18.7 (3)	N2—C17—C18—C19	−176.27 (18)
C3—C4—C8—O3	162.20 (19)	F1—C18—C19—C14	176.68 (17)
C5—C4—C8—O4	158.39 (17)	C17—C18—C19—C14	−2.6 (3)
C3—C4—C8—O4	−20.7 (3)	C15—C14—C19—C18	−2.4 (3)
C2—C3—C9—O5	17.1 (2)	C13—C14—C19—C18	178.29 (18)
C4—C3—C9—O5	−161.24 (18)	C17—N2—C20—C21	170.19 (18)
C2—C3—C9—O6	−158.98 (15)	C23—N2—C20—C21	−56.5 (2)
C4—C3—C9—O6	22.6 (3)	C22—N1—C21—C20	−56.8 (3)
C5—C6—C10—O7	−155.79 (18)	N2—C20—C21—N1	55.8 (3)
C1—C6—C10—O7	19.8 (3)	C21—N1—C22—C23	57.8 (3)
C5—C6—C10—O8	22.1 (2)	C17—N2—C23—C22	−168.47 (18)
C1—C6—C10—O8	−162.35 (16)	C20—N2—C23—C22	58.0 (2)
C15—N3—C11—C12	2.8 (3)	N1—C22—C23—N2	−58.2 (3)
C24—N3—C11—C12	−177.46 (18)	C11—N3—C24—C26	110.6 (2)
N3—C11—C12—C13	−0.7 (3)	C15—N3—C24—C26	−69.7 (2)
C11—C12—C13—O9	179.5 (2)	C11—N3—C24—C25	41.0 (3)
C11—C12—C13—C14	−0.7 (3)	C15—N3—C24—C25	−139.30 (18)
O9—C13—C14—C19	−0.9 (3)	N3—C24—C25—C26	106.74 (19)
C12—C13—C14—C19	179.23 (18)	N3—C24—C26—C25	−109.50 (19)
O9—C13—C14—C15	179.84 (18)		

### Hydrogen-bond geometry (Å, º)

**Table:**

*D*—H···*A*	*D*—H	H···*A*	*D*···*A*	*D*—H···*A*
O1—H1*C*···O3^i^	0.94	1.58	2.5170 (18)	177
O1—H1*C*···O4^i^	0.94	2.52	3.078 (2)	118
N1—H1*B*···O3^i^	0.93 (3)	2.54 (3)	3.006 (3)	111 (2)
N1—H1*B*···O1*W*^ii^	0.93 (3)	2.04 (3)	2.929 (3)	158 (2)
O8—H8*A*···O9^iii^	1.00	1.52	2.519 (2)	178.2

Symmetry codes: (i) *x*+1, *y*, *z*; (ii) −*x*+2, −*y*, −*z*+2; (iii) −*x*+2, −*y*, −*z*+1.
